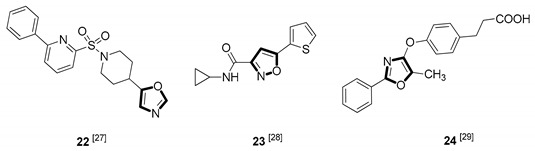# Recent Advances in the Synthesis of Oxazole-Based Molecules via van Leusen Oxazole Synthesis

**DOI:** 10.3390/molecules25071594

**Published:** 2020-03-31

**Authors:** Xunan Zheng, Wei Liu, Dawei Zhang

**Affiliations:** 1 College of Chemistry, Jilin University, Changchun 130012, China; zhengxn8217@mails.jlu.edu.cn; 2 College of Plant Science, Jilin University, Changchun 130062, China; 3 Department of Pesticide Science, Plant Protection College, Shenyang Agricultural University, Shenyang 110866, China

**Keywords:** van Leusen, TosMICs, oxazole, synthesis

## Abstract

Oxazole compounds, including one nitrogen atom and one oxygen atom in a five-membered heterocyclic ring, are present in various biological activities. Due to binding with a widespread spectrum of receptors and enzymes easily in biological systems through various non-covalent interactions, oxazole-based molecules are becoming a kind of significant heterocyclic nucleus, which have received attention from researchers globally, leading them to synthesize diverse oxazole derivatives. The van Leusen reaction, based on tosylmethylisocyanides (TosMICs), is one of the most appropriate strategies to prepare oxazole-based medicinal compounds. In this review, we summarize the recent advances of the synthesis of oxazole-containing molecules utilizing the van Leusen oxazole synthesis from 1972, aiming to look for potential oxazole-based medicinal compounds, which are valuable information for drug discovery and synthesis.

## 1. Introduction

The oxazole ring, with one nitrogen atom and one oxygen atom, which are widely displayed in natural products and synthetic molecules, is known as a prime skeleton for drug discovery. On the account of structural and chemical diversity, oxazole-based molecules, as a central scaffold, not only enable different types of interactions with various receptors and enzymes, showing broad biological activities, but also occupy a core position in medicinal chemistry, showing their enormous development value and they favored the discovery of newer potential therapeutic agents [1,2,3,4,5]. Consequently, a wide variety of oxazole-containing compounds, as clinical drugs or candidates, have been frequently employed, which play a vital role in the treatment of diverse types of diseases like antibacterial [6,7,8], antifungal [9,10,11], anti-inflammatory [12,13,14], antiviral [15,16,17], anti-tubercular [18,19,20], anticancer [21,22,23], anti-parasitic [24,25,26], antidiabetic [27,28,29], and so on. The marketed drugs containing the oxazole ring system with medicinal value are being actively exploited worldwide. Various pharmacological activities and chemical structures of oxazole-based molecules are enumerated in the following Table 1. 

Due to the diversity of therapeutic response profiles, the chemical synthesis of oxazole and its derivatives has become a key objective and has drawn much attention of current pharmacologists and chemists around the globe to be explored exhaustively for the benefit of mankind. Until now, many ingenious oxazole synthesis methodologies have been developed, including the van Leusen reaction [30], Cornforth reaction [31], Fisher reaction [32], Doyle reaction [33], Dakin–West reaction [34], as well as Robinson–Gabriel reaction [35], etc.. Among these synthetic strategies, it is well-known that the van Leusen oxazole synthesis, based on tosylmethylisocyanides (TosMICs), is one of the most convenient and attractive protocols for the preparation of oxazole-based molecules, owing to its excellent virtues like simple operation, easily obtained raw materials, and a broad substrate scope, and it has been developed rapidly in the past decades. It is worth mentioning that the pharmacological activity oxazole-based compounds **11**, **16** and **25** in Table 1 can be obtained by van Leusen reaction as a key step. 

**Scheme 1:**
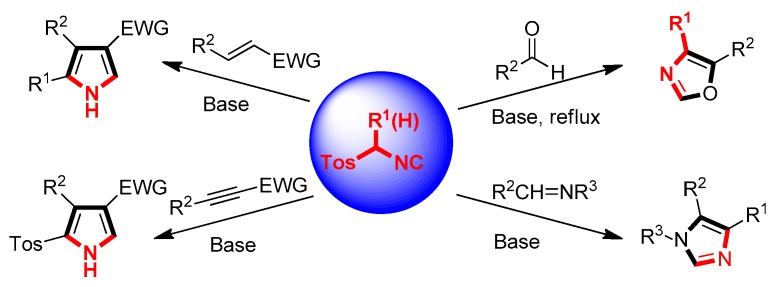
General van Leusen synthesis base on tosylmethylisocyanides (TosMICs).

TosMIC, a kind of the most significant reactants, has many good features at room temperature including stable solid, odorless, and colorless. Since it was introduced and applied in organic synthesis by the Dutch professor van Leusen in 1972, this reagent is also known as van Leusen’s reagent. Today, TosMIC and its derivatives have been recognized as one of the most significant building blocks in organic synthesis and a great deal of reaction scenarios, which have been fruitfully employed in the preparation of pyrrole-, imidazole-, and oxazole-based five-membered heterocyclic molecules [36,37,38,39,40]. General van Leusen synthesis base on TosMICs is summarized in Scheme 1.

Based on our previous research, we have published two reviews about the van Leusen reaction for the preparation of pyrrole- and imidazole-based molecules [41,42]. Therefore, this review, which covers the literature from 1972, will summarize the recent advances of the synthesis of oxazole-based molecules utilizing the van Leusen oxazole synthesis as an important part of van Leusen reaction, which is the [3+2] cycloaddition reaction based on TosMICs. Meanwhile, it is expected that this review article will be beneficial for fresh opportunities to search for a reasonable design for oxazole-containing drugs.

## 2. General van Leusen Oxazole Synthesis 

**Scheme 2:**
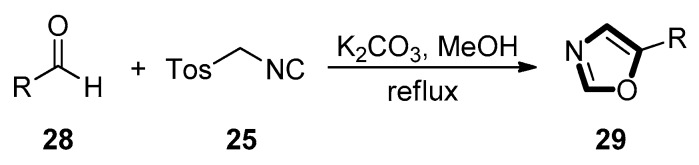
The first example of van Leusen oxazole synthesis.

In 1972, van Leusen et al. first discovered a novel chemical strategy for the formation of oxazole-based heterocyclic ring systems. In this study, 5-substituted oxazoles **29** were obtained from aldehydes **28** and TosMIC **25** as a precursor in a one-pot reaction under mild condition, which is widely known as the van Leusen oxazole synthesis (Scheme 2) [30]. 

**Scheme 3:**
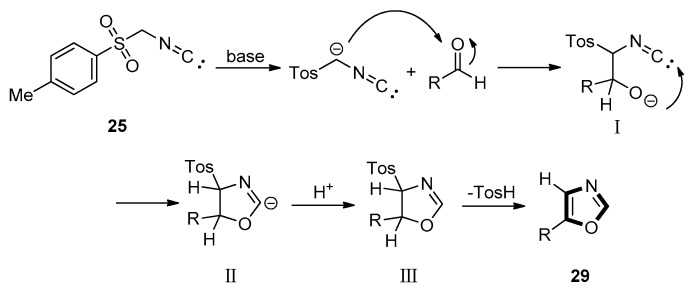
Mechanism of van Leusen oxazole synthesis.

As shown in Scheme 3, the van Leusen oxazole synthesis allows the preparation of 5- substituted oxazole through a two-step [3+2] cycloaddition reaction from aldehydes with TosMICs under a base condition. In this process, the TosMIC contains reactive isocyanide carbons, active methylene, and leaving groups as C2N1 “3-atom synthon”. After adding the deprotonated TosMIC to the aldehyde and bond formation between the resulting hydroxy group and the isocyano group, an oxazoline results as an intermediate. Then, with concomitant elimination of -TosH, the intermediates are given to the obtained 5-substituted oxazoles.

## 3. Developments of the van Leusen Oxazole Synthesis

**Scheme 4:**
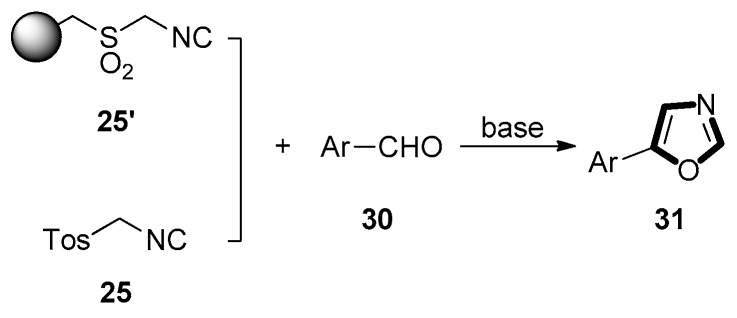
Synthesis of 5-aryloxazoles **31**.

In 1999, the Ganesan group developed a synthetic route of the 5-aryloxazoles **31**. They utilized a new polystyrene-SO_2_-CH_2_-NC with polystyrene-SH resin as starting material in three steps for the preparation of an immobilized sulfonylmethyl isocyanide **25’**, in order to illustrate the isocyanide resin’s potential. This protocol was similar to the solution-phase preparation of TosMIC or related analogues. Then, they used a quaternary ammonium hydroxide ion exchange resin to catalyze the reaction of TosMIC **25** with aromatic aldehydes **30** to generate the 5-aryloxazoles **31**. By analysis, they anticipated that polymer-supported-TosMIC (PS-TosMIC) and tetrabutylammonium hydroxide would be a realistic solid-phase replacement for other applications of TosMIC (Scheme 4) [43,44].

**Scheme 5:**
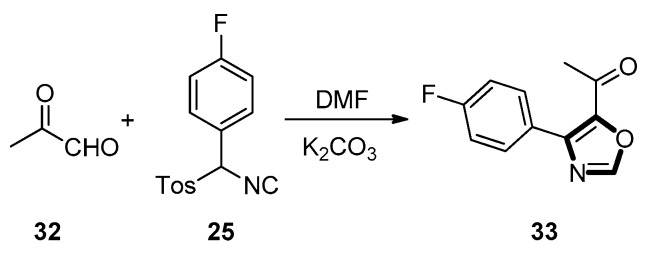
Synthesis of methylketone **33** via aryl-substituted TosMIC reagent.

In 2000, Sisko’s group found that van Leusen reaction of aryl-substituted TosMIC reagents with aldehydes had not been relatively explored. Hence, they surmised these reactions should be particularly concise, require mild reaction condition, and be tolerant of a vast range of functional groups. Then, they proved through the experiment that similar cycloaddition reactions with available and multifunctional aldehydes proceed smoothly to generate a wide variety of interesting oxazole compounds with high yield. For example, methylketone **33** could be obtained in a good yield from pyruvaldehyde **32** and aryl-substituted TosMIC reagent **25** (Scheme 5) [45].

**Scheme 6:**
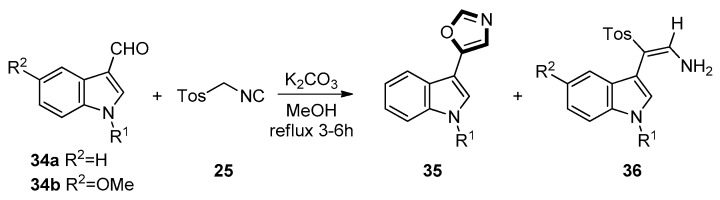
The reaction of 3/2-formylindoles with TosMIC and **34**.

**Scheme 7:**
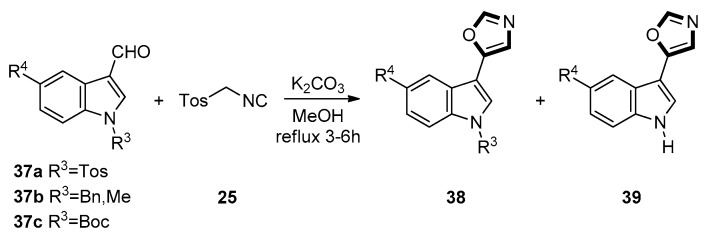
The reaction of 3/2-formylindoles with TosMIC and **37**.

In 2005, Chakrabarty et al. described a van Leusen route of 3/2-formylindoles with TosMIC to prepare corresponding indolyloxazoles and stable indolyl primary enamines. This reaction was provided in refluxing methanol and could complete quickly in 3–6 h. Besides the expected 5-(3-indolyl)oxazoles products, the novel rearranged indolyl primary enamines might be also formed. As shown in Scheme 6, two different results were obtained from substituted indole derivatives **34a** on one hand and from **34b** on the other hand. Specifically, each of **34a** furnished two products, viz. **35** and **36**, respectively. On the contrary, each of **34b** furnished only one type of products **36**. Likewise, as shown in Scheme 7, each of **37a** generated two products **38** and **39**, each of **37b** furnished only one product **38.** However, by testing **37c**, the result was that the final product was only **39** [46].

As to the mechanism of the formation of compound **36**, the intermediate products in the process of carboxylic acids through nitriles form aldehydes and ketones with TosMIC **25**. Then, the lone pair of electrons on the indolic nitrogen triggers the protonation (from methanol) of the enamidic double bond, resulting in the indoleninium species. Subsequently, a 1,2-shift of the tosyl group with simultaneous neutralization of the indoleninium cation, followed by the loss of a proton, gives rise to the intermediate. A nucleophilic attack by methanol, followed by the loss of a molecule of methyl formate and subsequent protonation, gives rise to the enamines **36**. 

**Scheme 8:**
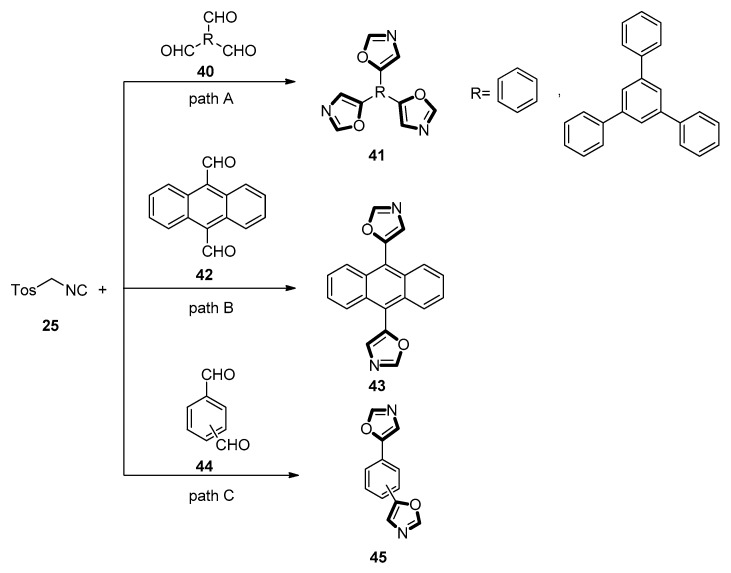
Synthesis of *C*_2_- and *C*_3_-symmetric oxazole derivatives **41**, **43**, and **45**.

In 2007, Kotha and co-workers reported that both *C*_2_-symmetric and *C*_3_-symmetric oxazole derivatives were synthesized through the stepwise Suzuki–Miyaura cross-coupling and the van Leusen synthesis. In this process, the raw materials of bis- and triscarboxaldehydes could be offered through a Suzuki–Miyaura cross-coupling reaction of corresponding arylboronic acid reagents. Then, the aldehydes were further treated with TosMIC **25** in the presence of K_2_CO_3_ in refluxing methanol to deliver the corresponding oxazole derivatives. The different products were generated surprisingly through changing the position and number of aldehyde groups. Specifically, when trialdehyde derivatives **40** were treated with TosMIC **25** in a typical reaction condition, the corresponding *C*_3_-symmetric oxazole derivatives **41** were generated (Scheme 8, path A). After confirming **41**, they also prepared other *C*_3_- and *C*_2_-symmetric oxazole derivatives **43** (Scheme 8, path B) and **45** through changing the substrates **42** and **44**, respectively (Scheme 8, path C) [47]. 

**Scheme 9:**
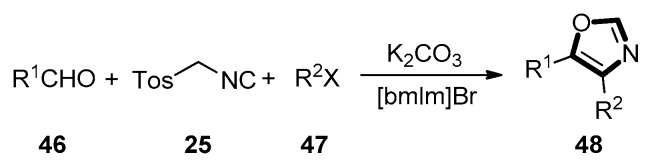
A one-pot synthesis of 4,5-disubstituted oxazoles **48** in ionic liquid.

In 2009, Yu and co-worker developed a van Leusen reaction to synthesize the 4,5-disubstituted oxazoles in a one-pot manner. The special characteristic of this reaction was the ionic liquid as solvent. The target oxazole products **48** were generated from TosMIC **25**, various aldehydes **46** and aliphatic halides **47**. This reaction had a high yield and a broad substrate scope. Since the electron withdrawing group could facilitate the reaction, aromatic aldehydes substrates with electron withdrawing group exhibited higher reactivity. It is noteworthy that this reaction is eco-friendly and economical, since the ionic liquids can be recovered and reused as solvent for six runs without conspicuous loss of yield (Scheme 9) [48]. 

From 2011 to 2018, the Šindler–Kulyk group reported a series of reactions to synthesize the 5-substituted oxazole compounds. 

**Scheme 10:**
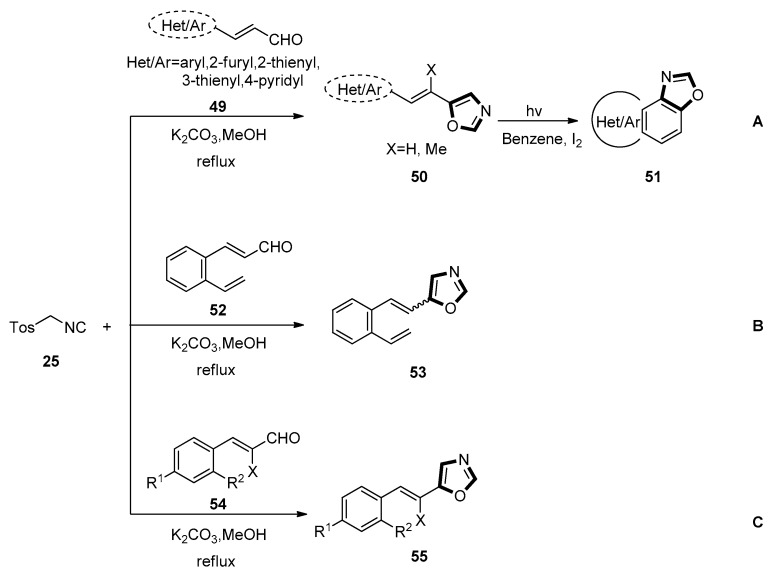
Synthesis of naphthoxazoles and fused heterobenzoxazoles **51**, trans-5-(2-vinylstyryl)oxazole **53**, and 5-(arylethenyl)oxazoles **55**.

In 2011, they described a novel approach for the synthesis of naphthoxazole and fused heterobenzoxazole derivatives. In this report, the starting 5-(aryl/furyl/thienyl/pyridyl ethenyl) oxazoles **50** were obtained through a van Leusen process between the corresponding *α*,*β*-unsaturated aldehydes **49** and TosMIC **25** in a good yield. Then, the intermediate oxazoles underwent UV irradiation in aerobic condition in the presence of iodine, transforming into the naphthoxazoles **51** and other fused heterobenzoxazoles (Scheme 10, method A) [49].

In 2014, they also synthesized the novel trans-5-(2-vinylstyryl)oxazole **53** through the van Leusen reaction between trans-3-(2-vinylphenyl) acrylaldehyde **52** and TosMIC **25**. The trans-3-(2-vinylphenyl) acrylaldehyde **52** was prepared through the Wittig reaction of ovinylbenzaldehyde and (formylmethylene) triphenylphosphorane. For the preparation of 5-(2-vinylstyryl) oxazole by this route, 3-(2-vinylphenyl) acrylaldehyde was needed. As the configuration of the starting aldehyde **52** for the van Leusen reaction with TosMIC **25** was in trans configuration, the obtained corresponding product 5-(2-vinylstyryl)oxazole **53** retained the trans configuration (Scheme 10, method B) [50].

The same group synthesized the required *p*- and *o*-substituted 5-arylethenyloxazoles **55** from the corresponding *α,β*-unsaturated aldehydes **54** and the TosMIC **25** through the van Leusen reaction under a typical reaction condition in 2018. To further broaden the substrate scope of naphtho [1–*d*]oxazoles, other 5-(arylethenyl)oxazoles **55** were prepared from the corresponding aryl-substituted *α*-unsaturated aldehydes **54** and TosMIC **25**. This process took place as depicted above, treating with TosMIC in one-step synthesis through the van Leusen reaction (Scheme 10, method C) [51]. 

**Scheme 11:**
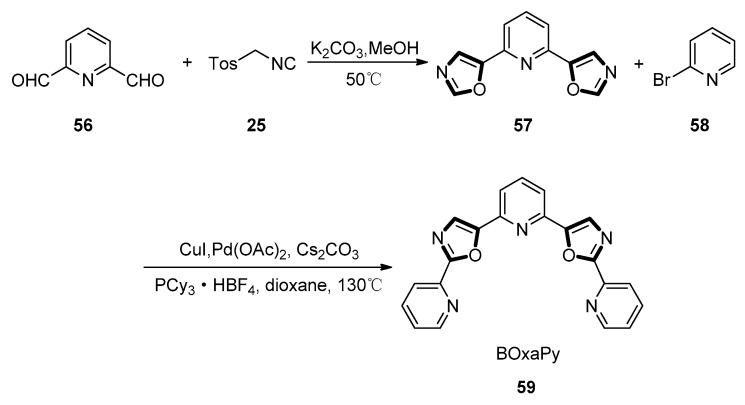
Synthesis of BOxaPy **59**.

**Scheme 12:**
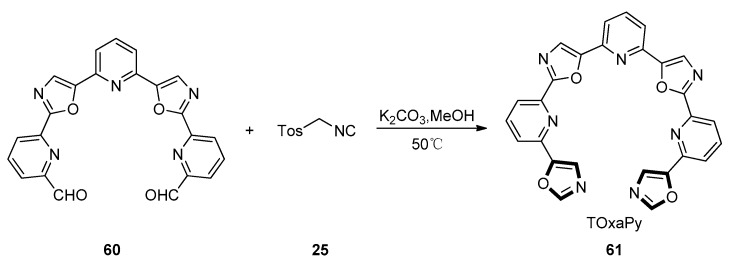
Synthesis of potential anticancer drug TOxaPy **61**.

In 2011, Hamon and co-workers adopted a convergent process based on the cross-coupling of 2,6-bis(oxazol-5-yl) pyridine **57** and 2-bromopyridine **58** derivatives through the double C-H activation of the oxazole rings, to perform the synthesis of oligo-heteroaryles. As shown in Scheme 11, precursor **57** was obtained under the van Leusen oxazole synthesis condition with TosMIC **25** and pyridine-2,6-dicarbaldehyde **56** as starting material which was afforded through the oxidation of 2,6-lutidine. The Pd catalyzed coupling of **57** and 2-bromopyridine **58** under known condition to form the pentaheteroaryle BOxaPy **59** in a 45% yield. To generate TOxaPy **61** unambiguously, another novel process was devised in which terminal oxazole moieties were synthesized from the corresponding pentacyclic bisformyl intermediate **60** (Scheme 12). The key step for the synthesis of TOxaPy **61** was the classic van Leusen oxazole synthesis between aldehyde **60** and TosMIC **25**, giving the target ligand TOxaPy **61** in a 50% yield. In this process, it is worth mentioning that the ligand TOxaPy **61** has an unprecedented binding behavior with quadruplexes, which shows a good potential for further application in the anticancer drug discovery [52]. 

**Scheme 13:**
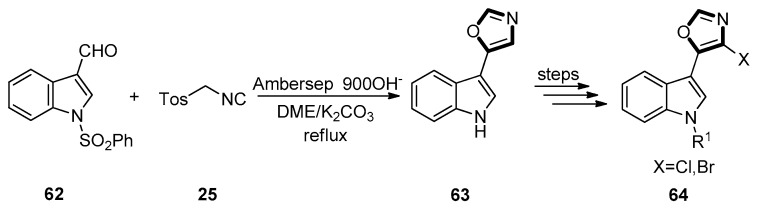
Synthesis of potential artificial compound **64**.

In 2012, Zhang et al. developed a straightforward and efficient synthetic method to form 5-(3-indolyl)-oxazoles **64**, further used to synthesize a class of products which were similar to the natural product pimprinine. The reaction, which was in a mixture of DME and methanol as solvent with TosMIC **25** from compound **62**, afforded 5-(1*H*-indol-3-yl)oxazole **63** in a 66% yield in the presence of the ion exchange resin of Ambersep^®^900(OH). By this way, they synthesized a sequence of pimprinine analogues and then tested their anti-fungal activities. The biological testing showed that some of the products display a broad spectrum anti-fungicidal activity against plant pathogens growing. At last, they found that seven of the products showing stronger activity were affirmed as the most prospective candidates for further study. Further structural optimization of pimprinine analogues was being well at present, alongside more precise biological testing of compounds with a high biological activity, in order to define their classes of fungicidal activity better (Scheme 13) [53].

**Scheme 14:**
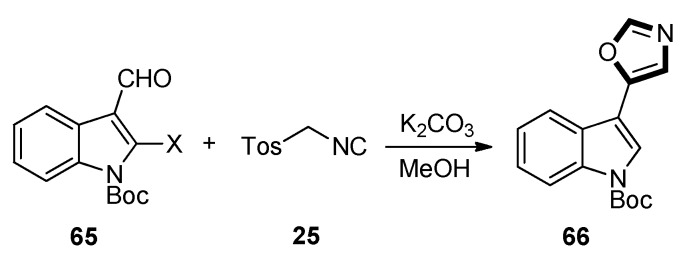
The marine natural product streptochlorin analogue **66**.

In 2013, Lee et al. published a protocol for the marine natural product streptochlorin. *N*-Boc protected aldehydes **65** were reacted with TosMIC **25** through a van Leusen reaction in the presence of K_2_CO_3_ as base in methanol, providing the desired oxazole compound **66**. It was interesting that the Boc protection group got lost during this transformation. To compensate for the undesired deprotection of the Boc protective group, it was reprotected at the *N*-1 position to give compound **66**. Then, further study was continued on this basis. In order to construct a series of streptochlorin-based, focused analog library, as well as the screening of additional bioactivities, researchers are now focusing on the further extension of the functional group tolerance and substrate scope of the above described synthetic sequences and the evaluation for additional bioactivities (Scheme 14) [54].

**Scheme 15:**
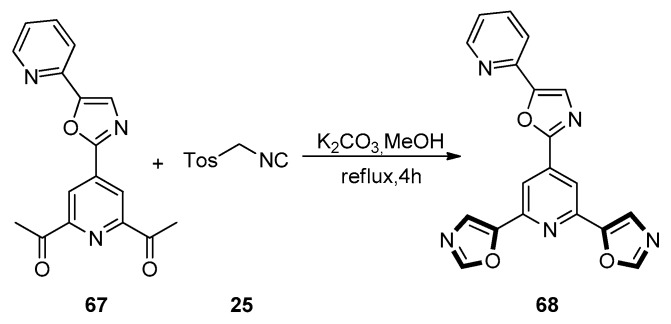
Synthesis of ‘propeller-like’ anticancer biomolecular target **68**.

In 2015, the Georgiades group reported a new synthetic route, which contained only seven steps, to synthesize the unusual ‘propeller-like’ pyridyl-oxazole architecture with alternating pyridines and oxazole rings. This method utilized the van Leusen reaction for the construction of oxazole moieties with aldehydes as starting materials, two Pd(II)/Cu(I)-mediated cross-coupling reactions including C-H activation of oxazoles for the construction of C-C bonds between bromopyridine intermediates and oxazole derivatives. As shown in Scheme 15, 1,3-dipolar cycloaddition progress was carried out on two ketone moieties of **67** with the reagent TosMIC **25**, under the condition of K_2_CO_3_ as base in refluxing methanol, making an efficient transformation of **67** into the target tris-oxazole compound **68** [55].

**Scheme 16:**
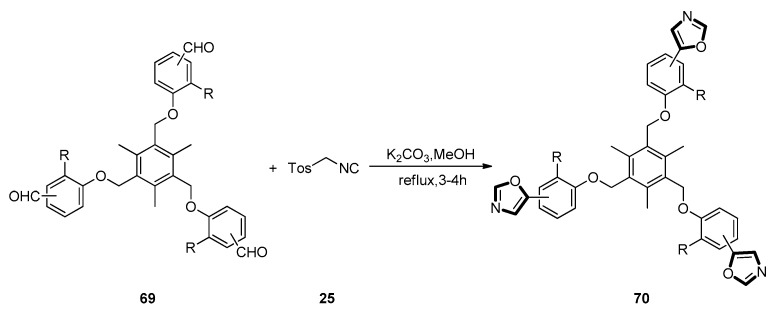
Synthesis of flexible tripodal 1,3-oxazoles **70** as molecular scaffolds.

In 2016, Shah et al. invented a preparation method of flexible tripodal 1,3-oxazoles through utilizing 1,3,5-trimethyl-benzene as a substrate for transformation into the *C*_3_-sym-metric scaffolds. The tris-aldehyde substrates were synthesized from 1,3,5-tris(bromomethyl)-2,4,6-trimethylbenzene through coupling with different phenolic aldehydes under a basic condition in a high yield. Then, the van Leusen reaction was used to construct the oxazole rings between tris-aldehyde substrates **69** and TosMIC **25** with K_2_CO_3_ in refluxing methanol, giving the desired 5-substituted tris-oxazoles **70** in a good yield (Scheme 16) [56].

**Scheme 17:**
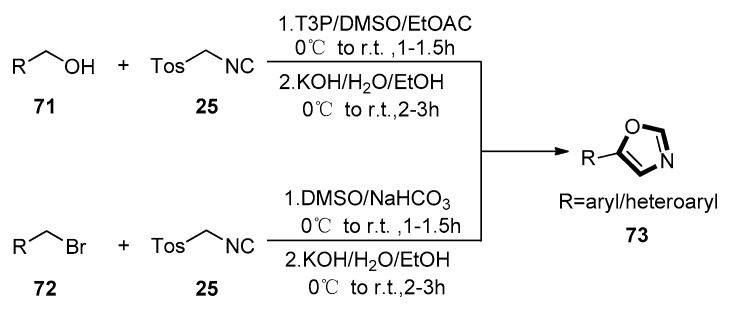
A novel modified van Leusen strategy for the synthesis of **73**.

In 2016, Vinary Kumar and co-workers described a novel modified van Leusen protocol for the preparation of 5-(het)aryl oxazoles **73**. They used substituted (het) aryl methyl alcohols **71** or benzyl bromides **72** as the starting materials which were converted to aldehyde through in-situ oxidation in a propylphosphonic anhydride (T3P^®^)–DMSO or DMSO media, respectively. Then, the homologous aldehyde reacted with TosMIC **25**, giving the oxazoles under the aqueous-alcoholic KOH condition with the yields ranging from 61% to 90%. This is the first substrate-modified van Leusen reaction which can proceed under a mild reaction condition, and it is tolerant of various functional groups with a high yield. This method is also efficient and eco-friendly, and the products are easily purified (Scheme 17) [57].

**Scheme 18:**
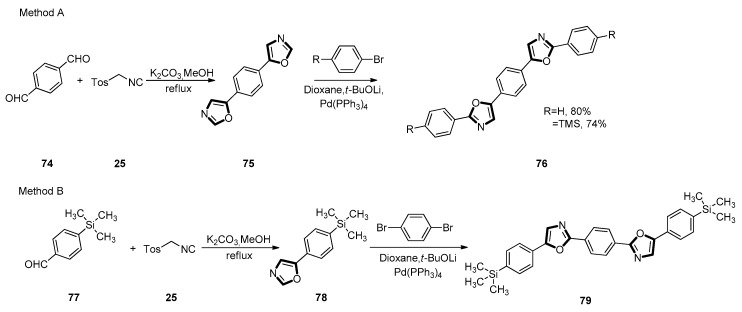
Synthesis of POPOP structural isomers **76** and **79**.

In the following year, the Ponomarenko group invented various synthetic routes for the preparation of linear phenyloxazoles and their trimethylsilane (TMS) group substituted derivatives, which could serve as the novel luminescent dye under a mild condition, by a combination of the van Leusen process and direct C-H arylation reaction. At the first step of method A, symmetric 1,4-bis(1,3-oxazol-5-yl)benzene **75** was generated from terephthaloyl aldehyde **74** and TosMIC **25** under the typical van Leusen condition. In the method B, 1-bromo-4-(trimethylsilyl)benzene was lithiated at the first step to obtain (trimethylsilyl)benzaldehyde **77**. After that, aldehyde **77** was translated into 5-substituted 4-(trimethylsilyl)phenyloxazole **78** with TosMIC **25** under the van Leusen condition. The target linear products **76** and **79**, were generated at the following steps by direct C-H arylation process with 4-substituted bromobenzene derivatives and 1,4-paradibromobenzene, with the yield of 80%, 74%, and 84%, respectively (Scheme 18).

The synthetic scheme of the two kinds of linear phenyloxazoles was exhibited, in which method A (Scheme 18) comprises fewer reaction stages compared with the preparation of the exact 1,4-bis(5-phenyloxazol-2-yl)benzene (POPOP) structure (Scheme 18, method B). Due to the concise and efficient synthesis and excellent optical properties as well as high photo- and thermo- stability, the novel luminescent dyes might find broad application in organic photonics [58].

**Scheme 19:**
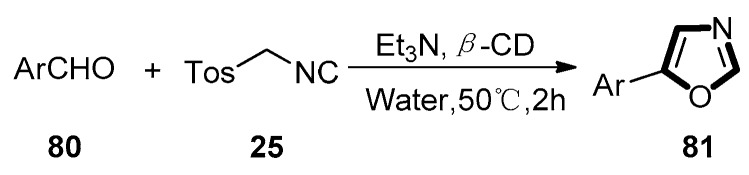
Synthesis of **81** by modified van Leusen reaction using green media.

In 2017, Mahdavi’s group described an efficient protocol for the formation of oxazoles. This approach proceeded through a unique van Leusen reaction in the presence of triethylamine and *β*-cyclodextrin (*β*-CD) with water as solvent. The reaction was formed between diverse aldehyde substrates **80** and TosMIC **25** in a system of Et_3_N and *β*-CD at 50 °C in water. It was clearly assured that the oxazole **81** was formed in an excellent yield through the analysis of the reaction mixtures. This novel approach improves the van Leusen reaction greatly since the catalytic amounts of base can be used to promote the reaction at low temperatures and water can be acted as a green media for this reaction (Scheme 19) [59].

**Scheme 20:**
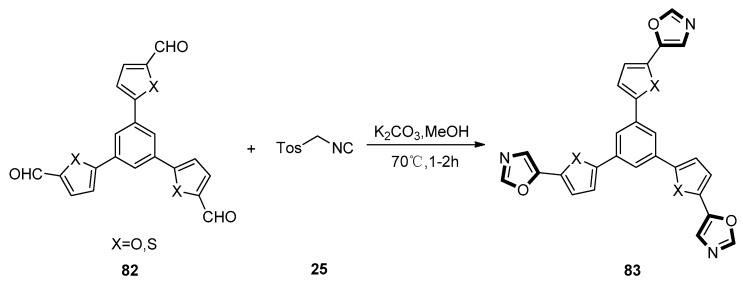
Synthesis of *C*_3_-symmetric star-shaped molecules **83**.

In 2017, Kotha and co-workers published a new application of a van Leusen synthesis. In this work, the van Leusen synthesis was used as a key step to construct a series of five-membered oxazole heterocycles, giving the star shaped molecules containing diverse heterocycles integrated with several variations **83**. The tri-aldehyde substrates **82** were synthesized from heterocycle derivatives through the Vilsmeier–Haack reaction under K_2_CO_3_ and methanol condition in a medium yield. This step had a broad substrate scope since the furan or thiophene derivatives could be synthesized equally. Then, the tri-aldehyde substrates **82** were treated with TosMIC **25**, transforming into the targeted oxazole products **83** through a van Leusen route (Scheme 20). In this work, the authors also investigated the fluorescent behavior of these molecules containing π-conjugated systems. The results indicated that the *C*_3_-Symmetric molecules containing furan moieties possessed a stronger fluorescence than thiophene-substituted star-shaped compounds [60].

**Scheme 21:**
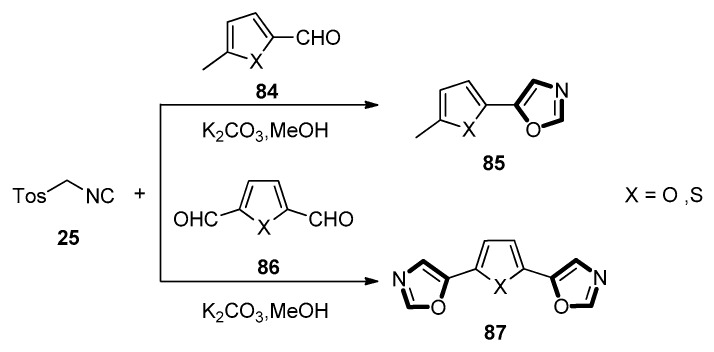
Synthesis of heterocyclic systems derivatives **85** and **87** based on TosMIC.

In 2017, Civcir et al. found a new application of the van Leusen reaction. In this work, a series of designed new molecules containing thiophene-based, furan-based oxazole, isoxazole, and isothiazole moieties were synthesized through the van Leusen reaction. By following this reaction, oxazole derivatives **85** with corresponding heterocyclic cores, were provided through the reaction of suitable 2-formyl substituted furan or thiophene derivatives **84** with TosMIC **25** (Scheme 21, top). Additionally, the three ring systems of furan or thiophene **87** which had the oxazole rings substituted **87** at their 2- and 5-positions, were also successfully obtained from the di-formyl substrates **86** (Scheme 21, bottom). This reaction was suitable for different aldehydes with furan or thiophene since both these oxazole synthetic methods occurred with good to excellent yield [61].

**Scheme 22:**
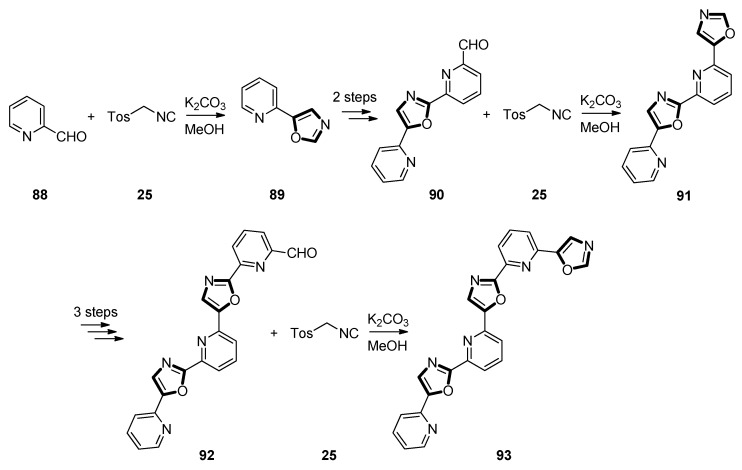
Synthesis of ‘head-to-tail’-connected asymmetric pyridyl-oxazoles **93** for anticancer purposes.

At the same year, the Georgiades group found that oligomeric chemical compounds composed by successive *N*,*O*-heteroaromatic rings, could be introduced for products with useful and mild properties as alternative substances for biomolecular recognition. Thereupon, they developed a synthetic method based on van Leusen reaction, along with oxazoles which were formed by C-H activation and following by their C-C cross-coupling to 2-bromopyridines in order to collect a class of variable-length, ‘head-to-tail’-connected, pyridyl-oxazole ligands. For constructing the skeleton of oligomers, one of the main chemical methods was to establish which was from the conversion of aldehyde moieties **88**, **90**, and **92** to oxazoles **89**, **91**, and **93** with TosMIC **25** in methanol, under stepwise van Leusen synthetic oxazole. The studies inspected the effect to link with asymmetry rather than symmetry of the ligands’ ingredient units, as well as the impact of ligand length, and results of these parameters, including target affinity, target stabilization, and target conformational modification. Through designing, these molecules representing new access into a series of rotationally flexible oligoaryl compounds, on account of limited rigidity and adaptability to the target surface and multivalent bonding ability, might be wonderfully suited to target sites of G-quadruplexes from those exploited by the more traditional intercalator-type ligands. Notably, they would focus on the basis of the potential function of these structures for anticancer purposes (Scheme 22) [62].

**Scheme 23:**
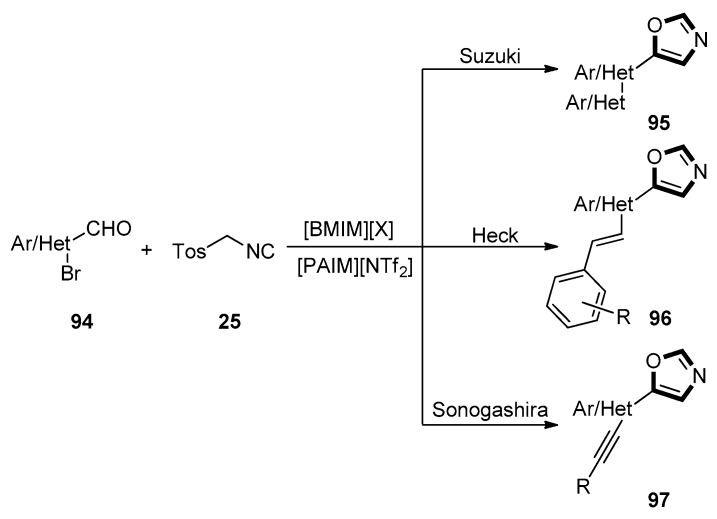
Synthesis of *C*_5_-substituted oxazoles **95**, **96**, and **97**.

In 2018, Laali reported that facile synthesis of multiple *C*_5_-substituted oxazoles derivatives **95**, **96**, and **97**. These products were obtained from the oxazole intermediates through the van Leusen reaction, followed by the sequential van Leusen–Suzuki, van Leusen–Heck, and van Leusen–Sonogashira coupling reactions, respectively. This method was characterized through employing imidazolium ionic liquids (imidazolium-ILs) as solvent and piperidine-appended imidazolium [PAIM][NTf_2_] as task-specific base. This reaction could proceed successfully with readily available aldehydes **94** and TosMIC **25** in a one-pot manner with a high yield. This reaction had great potential for the IL solvents which could be recycled and reused (Scheme 23) [63].

**Scheme 24:**
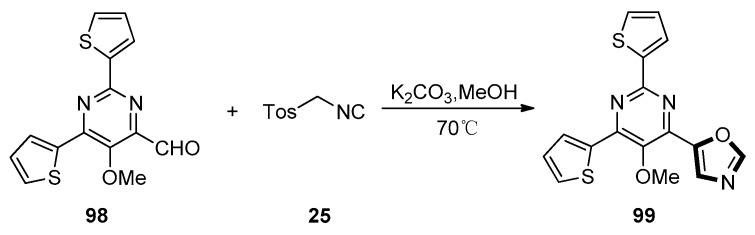
Synthesis of pyrimidine derivative **99**.

In 2019, Lechel et al. found a novel oxazole-functionalization of pyrimidine derivatives. In their study, they found that a van Leusen synthesis of **98** with TosMIC **25** could occur and give oxazole **99** in the K_2_CO_3_ and a methanol system in high yield. The synthesis of the novel pyrimidine derivative **99** containing three different heterocyclic substituents was unprecedented and remarkable, which indicated the flexibility and an application of this reaction in constructing the multi-substituted complex molecules (Scheme 24) [64].

**Scheme 25:**
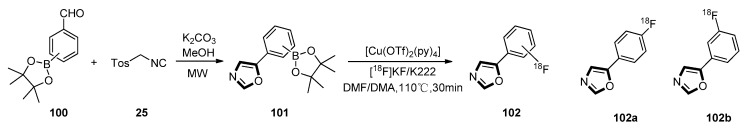
Synthesis of *p*- and *m*-position products **102**.

In the same year, Zarganes–Tzitzikas et al. developed the van Leusen reaction from formylphenylboronic ester **100**. They found that the widespread applicability of oxazoles in the preparation of drugs also justified the evaluation of the compatibility of these aromatic heterocycles with the Cu-mediated oxidative ^18^F-fluorination method. The oxazole substituted arylboronic ester derivatives **101** were formed through the van Leusen multicomponent reaction between a 4- or 3-formylphenylboronic ester **100** and TosMIC **25** under microwave assisted (MW-assisted) condition. The intermediates **101** were ^18^F-fluorinated through a Cu-mediated oxidative reaction. Both *p*- and *m*-position fluorinated molecules **102** were successfully radiolabeled. The 4-fluorinated product **102a** reached a higher maximum radiochemical conversion (RCC) of 67% than the 3-fluorinated product **102b** which had the maximum RCC of 38% (Scheme 25) [65]. 

**Scheme 26:**
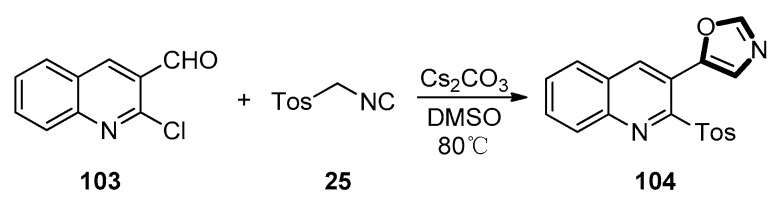
Synthesis of 5-(2-tosylquinolin-3-yl)oxazole **104**.

In 2019, Yasaei and co-workers described a synthesis of 5-(2-chloroquiolin-3-yl)oxazole **104**. The synthesis proceeded through a van Leusen route procedure between 2-chloroquinoline-3-carbaldehydes **103** and TosMIC **25**. The oxazole intermediate was efficiently subjected to the Pd-catalyzed amidation process with isocyanides to form 3-(oxazol-5-yl)quinoline-2-carboxamides. As shown in Scheme 26, subjecting 2-chloroquinoline-3-carbaldehyde **103** and TosMIC **25** to the van Leusen reaction condition generated 5-(2-tosylquinolin-3-yl)oxazole **104** in a 83% yield after 8 h (Scheme 26) [66].

**Scheme 27:**
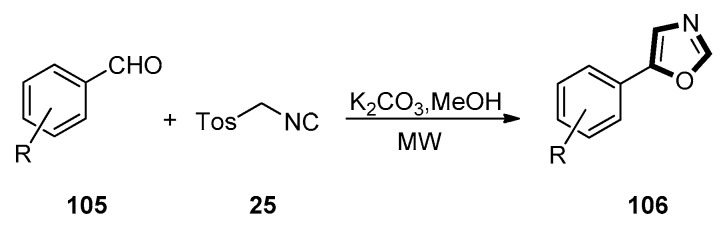
Synthesis of inhibitor of microbial pathogen **106**.

In 2020, Rashamuse et al. reported a MW-assisted van Leusen synthesis. They synthesized a series of unusual 5-aryl-4-tosyl-4,5-dihydro-1,3-oxazoles and 5-aryl-1,3-oxazole compounds **106** through a MW-assisted cycloaddition with TosMIC **25** and imines or aldehydes as starting materials. This reaction had both a high yield, efficiency, and a broad substrate scope. They extended the MW-assisted version of the van Leusen reaction of commercially available TosMIC and aldehyde to form of a small variety of 5-aryl-1,3-oxazoles **106** with anhydrous methanol as solvent (Scheme 27) [67].

## 4. Conclusions

In summary, under the in-depth research and application in oxazole-based medicinal chemistry and the progress in other related disciplines such as cell biology, molecular biology, pharmacology, and organic chemistry, the synthesis of oxazole-based drugs will be still an active field in medicinal research and development industries for a long time. In the future, increasing researcher interests would be focused on the design, synthesis, bioactive evaluation, and action mechanism of unusual types of oxazole-based heterocyclic compounds with completely novel chemical scaffold, which are helpful for overcoming drug resistances, increasing bioactivities, and will make remarkable contributions to the prevention and protection of human health. Hence, we could focus on changing the starting materials including different aldehydes and TosMICs to modify oxazole-containing derivatives, or utilizing a new synthesis technology like the MW-assisted condition to improve the synthesis efficiency, or uniting other typical name reaction to synthesize the special and complex oxazole-based compounds in the future. Above all, these have clearly and strongly suggested the infinite potentiality of van Leusen oxazole synthesis in medicinal chemistry. Additionally, we hope that this review will build a full foundation and reference source, which will open up new thought for researchers to focus on oxazole-based medicinal molecule design and synthesis chemistry.

## Figures and Tables

**Table 1:** The pharmacological activities and chemical structures of typical oxazole-based molecules.

Pharmacological Activities	Chemical Structures
Antibacterial	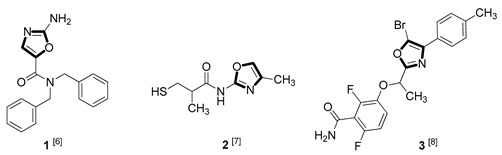
Antifungal	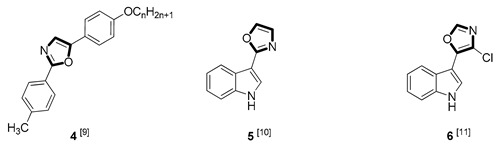
Anti-inflammatory	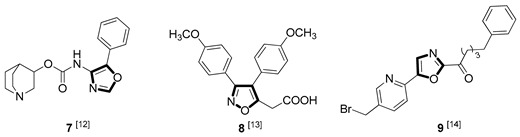
Antiviral	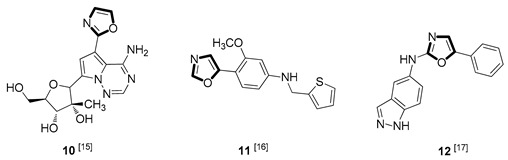
Antitubercular	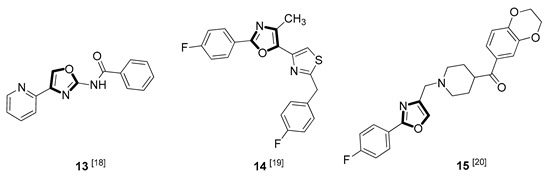
Anticancer	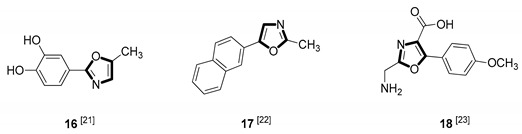
Antiparasitic	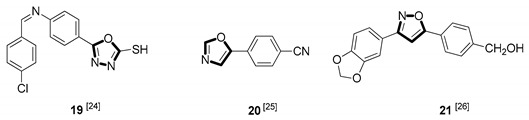
Antidiabetic